# 3-(4-Pyrid­yl)-4,5-dihydro-1*H*-benzo[*g*]indazole

**DOI:** 10.1107/S1600536808038944

**Published:** 2008-11-26

**Authors:** Xin Xiao, Yun-Qiang Zhang, Sai-Feng Xue, Qian-Jiang Zhu, Zhu Tao

**Affiliations:** a Key Laboratory of Macrocyclic and Supramolecular Chemistry of Guizhou Province, Guizhou University, Guiyang 550025, People’s Republic of China; b Institute of Applied Chemistry, Guizhou University, Guiyang 550025, People’s Republic of China

## Abstract

In the mol­ecular structure of the title compound, C_16_H_13_N_3_, the cyclo­hexa-1,3-diene ring displays a screw-boat conformation and the pyridine ring is twisted by a dihedral angle of 29.13 (9)° with respect to the pyrazole ring. Mol­ecules are linked into a supra­molecular structure by N—H⋯N hydrogen bonding.

## Related literature

For general background to indazole derivatives and their pharmacological properties, see: Bistochi *et al.* (1981 ▶); Keppler & Hartmann (1994 ▶); Sun *et al.* (1997 ▶); Gomtsyan *et al.* (2008 ▶).
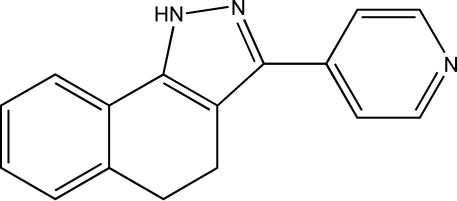


## Experimental

### 

#### Crystal data

C_16_H_13_N_3_
*M* *_r_* = 247.29
Orthorhombic, 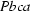

*a* = 15.306 (2) Å
*b* = 8.8368 (13) Å
*c* = 18.543 (3) Å
*V* = 2508.1 (6) Å^3^
*Z* = 8
Mo *K*α radiation
μ = 0.08 mm^−1^
*T* = 293 (2) K
0.22 × 0.19 × 0.18 mm

#### Data collection

Bruker SMART CCD area-detector diffractometer
Absorption correction: none
24926 measured reflections
2217 independent reflections
1978 reflections with *I* > 2σ(*I*)
*R*_int_ = 0.036

#### Refinement

*R*[*F* ^2^ > 2σ(*F* ^2^)] = 0.048
*wR*(*F* ^2^) = 0.130
*S* = 1.08
2217 reflections
172 parameters
H-atom parameters constrained
Δρ_max_ = 0.44 e Å^−3^
Δρ_min_ = −0.25 e Å^−3^

### 

Data collection: *SMART* (Bruker, 2002 ▶); cell refinement: *SAINT* (Bruker, 2002 ▶); data reduction: *SAINT*; program(s) used to solve structure: *SHELXS97* (Sheldrick, 2008 ▶); program(s) used to refine structure: *SHELXL97* (Sheldrick, 2008 ▶); molecular graphics: *ORTEP-3 for Windows* (Farrugia, 1997 ▶); software used to prepare material for publication: *WinGX* (Farrugia, 1999 ▶).

## Supplementary Material

Crystal structure: contains datablocks global, I. DOI: 10.1107/S1600536808038944/xu2463sup1.cif

Structure factors: contains datablocks I. DOI: 10.1107/S1600536808038944/xu2463Isup2.hkl

Additional supplementary materials:  crystallographic information; 3D view; checkCIF report

## Figures and Tables

**Table 1:** Hydrogen-bond geometry (Å, °)

*D*—H⋯*A*	*D*—H	H⋯*A*	*D*⋯*A*	*D*—H⋯*A*
N2—H2⋯N3^i^	0.86	2.17	2.895 (2)	141

Symmetry code: (i) 
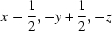
.

**Fig. 1.**
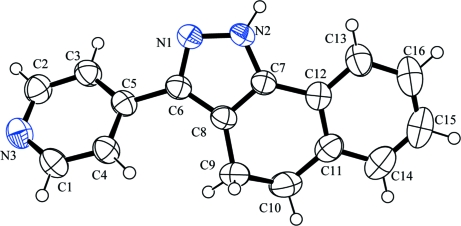
The molecular structure of (I) showing the atom-labeling scheme. Displacement ellipsoids are drawn at the 50% probability level.

## supplementary crystallographic information

### Comment

Indazole derivatives exhibit variety of pharmacological properties such as anti-inflammatory (Bistochi *et al.*, 1981), antitumor (Keppler *et al.*, 1994), anti-HIV (Sun *et al.*, 1997) and analgesic (Gomtsyan *et al.*, 2008). Herein we present the crystal structure of the title indazole derivative.

The crystal structure of the title compound is represented in Fig. 1. the C10-containing cyclohexa-1,3-diene ring displays a screw-boat conformation, and the pyridine ring is twisted to pyrazole ring with a dihedral angle of 29.13 (9)°. Intermolecular N—H···N hydrogen bonding presents in the crystal structure (Table 1).

### Experimental

A solution of 3,4-dihydronaphthalen-1(2*H*)-one (1.46 g, 0.01 mol) was added to a stirred solution of hydrazine (0.05 g, 0.01 mol) in dry tetrahydrofuran (50 ml) at 273 K for 3 h. Then n-butyllithium (0.02 mol) was added at a fast dropwise rate during a 5 min period at 273 K. The solution was stirred at 273 K for an additional 30 min, then methyl isonicotinate (1.37 g, 0.01 mol) dissolved in 40 ml of THF was added to the dilithiated intermediate, and the solution was stirred for 1 h at 273 K. Finally, 20 ml of 3 *M* hydrochloric acid was added, and the two phase mixture was well stirred and heated under reflux for 45 min. The mixture was then neutralized with solid sodium bicarbonate, and the layers were separated. The aqueous layer was extracted with ether. The organic fractions were combined, evaporated, the crude product was dissolved in methanol (60 ml). The solution was filtered and the filtrate was set aside for three weeks to obtain colorless single crystals of the title compound.

### Refinement

H atoms were placed in calculated positions with C—H = 0.93 or 0.97 Å, N—H = 0.86 Å, and refined in riding mode with U_iso_(H) = 1.2U_eq_(C,N).

### Crystal data

**Table:**

C_16_H_13_N_3_	*F*_000_ = 1040
*M**_r_* = 247.29	*D*_x_ = 1.310 Mg m^−^^3^
Orthorhombic, *P**b**c**a*	Mo *K*α radiation λ = 0.71073 Å
Hall symbol: -P 2ac 2ab	Cell parameters from 2217 reflections
*a* = 15.306 (2) Å	θ = 2.2–25.0º
*b* = 8.8368 (13) Å	µ = 0.08 mm^−^^1^
*c* = 18.543 (3) Å	*T* = 293 (2) K
*V* = 2508.1 (6) Å^3^	Block, colorless
*Z* = 8	0.22 × 0.19 × 0.18 mm

### Data collection

**Table:**

Bruker SMART CCD area-detector diffractometer	1978 reflections with *I* > 2σ(*I*)
Radiation source: fine-focus sealed tube	*R*_int_ = 0.036
Monochromator: graphite	θ_max_ = 25.0º
*T* = 293(2) K	θ_min_ = 2.2º
φ and ω scans	*h* = −18→17
Absorption correction: none	*k* = −10→9
24926 measured reflections	*l* = −22→22
2217 independent reflections	

### Refinement

**Table:**

Refinement on *F*^2^	Secondary atom site location: difference Fourier map
Least-squares matrix: full	Hydrogen site location: inferred from neighbouring sites
*R*[*F*^2^ > 2σ(*F*^2^)] = 0.048	H-atom parameters constrained
*wR*(*F*^2^) = 0.130	*w* = 1/[σ^2^(*F*_o_^2^) + (0.0626*P*)^2^ + 0.7587*P*] where *P* = (*F*_o_^2^ + 2*F*_c_^2^)/3
*S* = 1.08	(Δ/σ)_max_ < 0.001
2217 reflections	Δρ_max_ = 0.44 e Å^−^^3^
172 parameters	Δρ_min_ = −0.25 e Å^−^^3^
Primary atom site location: structure-invariant direct methods	Extinction correction: none

### Special details

**Table:**

Geometry. All e.s.d.'s (except the e.s.d. in the dihedral angle between two l.s. planes) are estimated using the full covariance matrix. The cell e.s.d.'s are taken into account individually in the estimation of e.s.d.'s in distances, angles and torsion angles; correlations between e.s.d.'s in cell parameters are only used when they are defined by crystal symmetry. An approximate (isotropic) treatment of cell e.s.d.'s is used for estimating e.s.d.'s involving l.s. planes.
Refinement. Refinement of *F*^2^ against ALL reflections. The weighted *R*-factor *wR* and goodness of fit *S* are based on *F*^2^, conventional *R*-factors *R* are based on *F*, with *F* set to zero for negative *F*^2^. The threshold expression of *F*^2^ > σ(*F*^2^) is used only for calculating *R*-factors(gt) *etc*. and is not relevant to the choice of reflections for refinement. *R*-factors based on *F*^2^ are statistically about twice as large as those based on *F*, and *R*- factors based on ALL data will be even larger.

### Fractional atomic coordinates and isotropic or equivalent isotropic displacement parameters (Å^2^)

**Table:**

	*x*	*y*	*z*	*U*_iso_*/*U*_eq_	
N2	0.37603 (9)	0.33266 (16)	0.11204 (7)	0.0442 (4)	
H2	0.3299	0.3882	0.1124	0.053*	
N1	0.43088 (9)	0.32191 (17)	0.05503 (8)	0.0458 (4)	
C2	0.65724 (12)	0.2397 (2)	−0.07332 (10)	0.0563 (5)	
H2A	0.6747	0.3078	−0.1087	0.068*	
C11	0.41088 (14)	0.1591 (2)	0.29215 (10)	0.0567 (5)	
C5	0.56409 (10)	0.18489 (19)	0.02662 (9)	0.0436 (4)	
C7	0.40188 (10)	0.24622 (18)	0.16853 (9)	0.0415 (4)	
C12	0.36103 (11)	0.2337 (2)	0.23932 (9)	0.0466 (4)	
C8	0.47774 (11)	0.17516 (19)	0.14790 (9)	0.0438 (4)	
C6	0.49343 (10)	0.22560 (19)	0.07685 (9)	0.0425 (4)	
C4	0.60463 (11)	0.0446 (2)	0.02934 (10)	0.0484 (4)	
H4	0.5881	−0.0261	0.0639	0.058*	
C9	0.52238 (13)	0.0675 (2)	0.19845 (11)	0.0631 (5)	
H9A	0.5018	−0.0346	0.1895	0.076*	
H9B	0.5849	0.0696	0.1899	0.076*	
C1	0.66954 (11)	0.0109 (2)	−0.01959 (10)	0.0534 (5)	
H1	0.6958	−0.0838	−0.0165	0.064*	
N3	0.69715 (9)	0.10469 (18)	−0.07098 (8)	0.0534 (4)	
C3	0.59220 (12)	0.2840 (2)	−0.02683 (10)	0.0528 (5)	
H3	0.5672	0.3795	−0.0311	0.063*	
C13	0.27959 (13)	0.2938 (2)	0.25682 (11)	0.0614 (5)	
H13	0.2468	0.3434	0.2219	0.074*	
C14	0.37630 (18)	0.1460 (3)	0.36132 (11)	0.0726 (6)	
H14	0.4082	0.0962	0.3968	0.087*	
C15	0.29537 (18)	0.2057 (3)	0.37810 (12)	0.0771 (7)	
H15	0.2733	0.1959	0.4246	0.092*	
C16	0.24751 (16)	0.2795 (3)	0.32620 (12)	0.0761 (7)	
H16	0.1932	0.3200	0.3378	0.091*	
C10	0.50413 (16)	0.1100 (3)	0.27516 (12)	0.0759 (7)	
H10A	0.5432	0.1918	0.2884	0.091*	
H10B	0.5183	0.0240	0.3055	0.091*	

### Atomic displacement parameters (Å^2^)

**Table:**

	*U*^11^	*U*^22^	*U*^33^	*U*^12^	*U*^13^	*U*^23^
N2	0.0354 (7)	0.0488 (8)	0.0484 (8)	0.0074 (6)	−0.0015 (6)	0.0005 (6)
N1	0.0395 (8)	0.0497 (8)	0.0480 (8)	0.0035 (6)	−0.0009 (6)	0.0016 (6)
C2	0.0498 (11)	0.0611 (12)	0.0579 (11)	−0.0071 (9)	0.0052 (8)	0.0048 (9)
C11	0.0704 (12)	0.0498 (11)	0.0499 (10)	−0.0028 (9)	−0.0010 (9)	0.0031 (8)
C5	0.0331 (8)	0.0480 (9)	0.0496 (9)	−0.0036 (7)	−0.0023 (7)	−0.0032 (7)
C7	0.0375 (9)	0.0422 (9)	0.0447 (9)	−0.0016 (7)	−0.0033 (7)	−0.0008 (7)
C12	0.0474 (10)	0.0449 (9)	0.0474 (9)	−0.0077 (8)	0.0013 (7)	−0.0040 (7)
C8	0.0379 (9)	0.0430 (9)	0.0504 (10)	0.0011 (7)	−0.0024 (7)	0.0020 (7)
C6	0.0361 (8)	0.0416 (9)	0.0498 (9)	−0.0019 (7)	−0.0022 (7)	0.0007 (7)
C4	0.0412 (9)	0.0455 (10)	0.0585 (10)	−0.0024 (7)	0.0045 (8)	0.0002 (8)
C9	0.0590 (12)	0.0645 (12)	0.0658 (12)	0.0145 (10)	−0.0004 (9)	0.0158 (10)
C1	0.0428 (10)	0.0493 (10)	0.0681 (12)	−0.0001 (8)	0.0046 (8)	−0.0068 (9)
N3	0.0396 (8)	0.0601 (10)	0.0605 (9)	−0.0063 (7)	0.0053 (7)	−0.0069 (7)
C3	0.0455 (10)	0.0520 (11)	0.0610 (11)	0.0020 (8)	0.0051 (8)	0.0058 (9)
C13	0.0494 (11)	0.0756 (13)	0.0592 (11)	−0.0032 (10)	0.0058 (9)	−0.0080 (10)
C14	0.0972 (18)	0.0692 (14)	0.0515 (11)	−0.0096 (13)	0.0013 (11)	0.0068 (10)
C15	0.0888 (17)	0.0870 (16)	0.0554 (12)	−0.0268 (14)	0.0208 (12)	−0.0080 (11)
C16	0.0628 (13)	0.0966 (17)	0.0689 (13)	−0.0122 (12)	0.0210 (11)	−0.0184 (13)
C10	0.0849 (14)	0.0828 (16)	0.0600 (12)	0.0256 (12)	−0.0095 (11)	0.0120 (11)

### Geometric parameters (Å, °)

**Table:**

N2—N1	1.353 (2)	C4—C1	1.378 (2)
N2—C7	1.356 (2)	C4—H4	0.9300
N2—H2	0.8600	C9—C10	1.497 (3)
N1—C6	1.343 (2)	C9—H9A	0.9700
C2—N3	1.341 (3)	C9—H9B	0.9700
C2—C3	1.374 (3)	C1—N3	1.332 (2)
C2—H2A	0.9300	C1—H1	0.9300
C11—C14	1.393 (3)	C3—H3	0.9300
C11—C12	1.406 (3)	C13—C16	1.383 (3)
C11—C10	1.525 (3)	C13—H13	0.9300
C5—C4	1.387 (2)	C14—C15	1.382 (4)
C5—C3	1.391 (2)	C14—H14	0.9300
C5—C6	1.472 (2)	C15—C16	1.374 (4)
C7—C8	1.374 (2)	C15—H15	0.9300
C7—C12	1.458 (2)	C16—H16	0.9300
C12—C13	1.393 (3)	C10—H10A	0.9700
C8—C6	1.412 (2)	C10—H10B	0.9700
C8—C9	1.500 (2)		
			
N1—N2—C7	112.52 (13)	C8—C9—H9A	109.6
N1—N2—H2	123.7	C10—C9—H9B	109.6
C7—N2—H2	123.7	C8—C9—H9B	109.6
C6—N1—N2	104.56 (13)	H9A—C9—H9B	108.1
N3—C2—C3	124.28 (18)	N3—C1—C4	124.42 (18)
N3—C2—H2A	117.9	N3—C1—H1	117.8
C3—C2—H2A	117.9	C4—C1—H1	117.8
C14—C11—C12	118.3 (2)	C1—N3—C2	115.61 (16)
C14—C11—C10	121.50 (19)	C2—C3—C5	119.47 (18)
C12—C11—C10	119.84 (17)	C2—C3—H3	120.3
C4—C5—C3	116.76 (16)	C5—C3—H3	120.3
C4—C5—C6	121.60 (16)	C16—C13—C12	120.0 (2)
C3—C5—C6	121.63 (16)	C16—C13—H13	120.0
N2—C7—C8	106.80 (14)	C12—C13—H13	120.0
N2—C7—C12	127.82 (15)	C15—C14—C11	121.1 (2)
C8—C7—C12	125.33 (15)	C15—C14—H14	119.5
C13—C12—C11	120.13 (17)	C11—C14—H14	119.5
C13—C12—C7	124.38 (17)	C16—C15—C14	120.1 (2)
C11—C12—C7	115.47 (16)	C16—C15—H15	120.0
C7—C8—C6	105.03 (14)	C14—C15—H15	120.0
C7—C8—C9	120.04 (16)	C15—C16—C13	120.4 (2)
C6—C8—C9	134.91 (16)	C15—C16—H16	119.8
N1—C6—C8	111.09 (15)	C13—C16—H16	119.8
N1—C6—C5	119.20 (15)	C9—C10—C11	116.24 (18)
C8—C6—C5	129.68 (15)	C9—C10—H10A	108.2
C1—C4—C5	119.47 (17)	C11—C10—H10A	108.2
C1—C4—H4	120.3	C9—C10—H10B	108.2
C5—C4—H4	120.3	C11—C10—H10B	108.2
C10—C9—C8	110.47 (17)	H10A—C10—H10B	107.4
C10—C9—H9A	109.6		

### Hydrogen-bond geometry (Å, °)

**Table:**

*D*—H···*A*	*D*—H	H···*A*	*D*···*A*	*D*—H···*A*
N2—H2···N3^i^	0.86	2.17	2.895 (2)	141

Symmetry codes: (i) *x*−1/2, −*y*+1/2, −*z*.

## References

[bb1] Bistochi, G. A., De Meo, G., Pedini, M., Ricci, A., Brouilhet, H., Bucherie, S., Rabaud, M. & Jacquignon, P. (1981). *Farmaco Ed. Sci.* **36**, 315–333. 7238851

[bb2] Bruker (2002). *SMART* and *SAINT* Bruker AXS, Inc., Madison, Wisconsin, USA.

[bb3] Farrugia, L. J. (1997). *J. Appl. Cryst.* **30**, 565.

[bb4] Farrugia, L. J. (1999). *J. Appl. Cryst.* **32**, 837–838.

[bb5] Gomtsyan, A., Bayburt, E. K., Schmidt, R. G., Surowy, C. S., Honore, P., Marsh, K. C., Hannick, S. M., McDonald, H. A., Wetter, J. M., Sullivan, J. P., Jarvis, M. F., Faltynek, C. R. & Lee, C. H. (2008). *J. Med. Chem.* **51**, 392–395. 10.1021/jm701007g 18183945

[bb6] Keppler, B. K. & Hartmann, M. (1994). *Met. Based Drugs.* **1**, 145–149. 10.1155/MBD.1994.145 PMC2364891 18476225

[bb7] Sheldrick, G. M. (2008). *Acta Cryst.* A**64**, 112–122. 10.1107/S0108767307043930 18156677

[bb8] Sun, J. H., Teleha, C. A., Yan, J. S., Rodgers, J. D. & Nugiel, D. A. (1997). *J. Org. Chem.* **62**, 5627–5629.

